# Consanguineous marriages in the genetic counseling centers of Isfahan and the ethical issues of clinical consultations

**Published:** 2017-12-10

**Authors:** Narges Nouri, Nayereh Nouri, Samane Tirgar, Elham Soleimani, Vida Yazdani, Farzaneh Zahedi, Bagher Larijani

**Affiliations:** 1 *Tohid Genetic Counseling Center, Isfahan, Iran.*; 2 *Genetic Laboratory of Al- Zahra Hospital, Isfahan University of Medical Sciences, Isfahan, Iran.*; 3 *Endocrinology and Metabolism Research Center, Endocrinology and Metabolism Clinical Sciences Institute, Tehran University of Medical Sciences, Tehran, Iran; Faculty of Sciences, Isfahan University, Isfahan, Iran. *; 4 *Medical Genetics Laboratory of Genome, Isfahan, Iran. *; 5 *Endocrinology and Metabolism Research Center, Endocrinology and Metabolism Clinical Sciences Institute, Tehran University of Medical Sciences, Tehran, Iran. *; 6 *Professor, Medical Ethics and History of Medicine Research Center, Tehran University of Medical Sciences, Tehran, Iran.*

**Keywords:** *Genetic counseling*, *Genetics*, *Consanguineous marriage*, *Medical ethics*, *Iran*

## Abstract

Consanguineous marriage, which is common in many regions in the world, has absorbed much attention as a causative factor in raising the incidence of genetic diseases. The adverse effects may be attributed to the expression of the genes received from common ancestors and mortality and morbidity of the offspring. Iran has a high rate of consanguineous marriages. In recent years genetic counseling has come to be considered in health care services. This cross-sectional study was conducted in order to determine the prevalence and types of consanguineous marriages in the genetic clinics in Isfahan. We aimed to define the different types of marriages, specific categories of genetic disorders associated with consanguineous marriages, and mode of inheritance in the family tree. We also narratively reviewed the ethical aspects of the issue.

The data were collected using a simple questionnaire. A total number of 1535 couples from urban and rural areas formed the study population. The marriages were classified according to the degree of the relationship between couples, including: double cousin, first cousin, first cousin once removed, second cousin and beyond second cousin. The SPSS software version 16 was used for data analysis.

Data obtained through genetic counseling offered during a 5-year period revealed that 74.3% had consanguineous relationships, 62.3% were first cousins, 1% were double cousins and 7.8% were second cousins. In addition, 76% of the couples had at least one genetic disease in their family tree. Related ethical issues were also considered in this study, including autonomy and informed decision making, benefit and harm assessment, confidentiality, ethics in research, justice in access to counseling services, financial problems ethics, and the intellectual property of scientific success.

## Introduction

Nowadays, the infant mortality rate has significantly declined due to the improvement in health and nutrition care and also the control of infectious diseases. However, one handicapped child is born every 8 minutes, of which 90% are in the developing countries (1-3). Consanguineous marriage has been recognized as one of the important causes of these disorders (1, 4-7). These facts point out the necessity of training and preventive counseling with regard to consanguineous marriages.

Consanguineous marriages take place between blood relatives, that is, the husband and wife should have at least one common ancestor (1, 6). These individuals are first cousins (e.g. daughter of paternal uncle, daughter of maternal aunt, daughter of maternal uncle, etc.); second cousins (e.g. grandchild of paternal uncle, grandchild of maternal aunt, grandchild of paternal aunt, etc.); third cousins (e.g. great grandchild of maternal aunt, grandchild of half aunt, grandchild of maternal aunt, etc.); and double cousins (e.g. bilateral parallel cousins). The risk of having babies with congenital diseases in consanguineous marriages is 2 - 3 times higher than non-consanguineous ones (8-12).

The frequency of consanguineous marriages varies between less than 1% in North America, Europe, Russia, and Australia, and more than 50% in some Arab countries, Turkey, Iran, Pakistan, and South India (9, 13).

Previous studies have shown that this type of marriage is more common in Islamic and Arab countries, having a heterogeneous prevalence depending on custom, culture, religion, and the geographical zones within these countries (9, 13-16). One-fifth of the world’s population, namely about 1.1 billion people, lives in countries where one in every three marriages on average is a consanguineous marriage (1-3, 9, 11, 14, 17).

In Islam, there is a great emphasis on marriage and child support, and sufficient advice is given concerning marriage, choosing a spouse, the relationship between couples and their duties and rights, as well as protection of children and their rights (18, 19). Consanguineous marriage has not been recommended, but rather criticized by Islam. The hadiths of the Infallibles advise against consanguineous marriage; as a case in point, Prophet Muhammad (PBUH) has been quoted to say, “Marry strangers to have no weak children”1. There is also a quote by Imam Sadegh (PBUH), the 6^th^ leader (*Emam*) of Shia Muslims, saying, “Do not marry blood relatives, because weak babies will be born”2. Historical evidence shows that only two of the fourteen Infallible Shia leaders have had consanguineous marriages (18, 19).

By the end of the twentieth century, many researchers believed that the prevalence of consanguineous marriage would decline due to the industrialization and the increasing educational achievements of women. However, in most geographical areas, consanguineous marriage has increased or remained unchanged (9, 13, 17, 20). In Iran, the statistics available on some parts of the country have shown different results concerning the frequency of consanguineous marriages. According to a study conducted on 300,000 Iranian couples from different ethnic groups in Iran, more than 38% of the marriages were consanguineous, of which 70% were between first cousins (21). In another study in Tehran, marriages in three successive generations were studied and the results showed that consanguineous marriages in the third generation had significantly increased in comparison to the first and second generations (22).

Billions of dollars are spent every year to treat and control the congenital diseases resulting from consanguineous marriages. In Arab countries, the treatment of 4 hereditary diseases including thalassemia, sickle-cell anemia, cystic fibrosis and hemophilia costs more than 13 billion dollars annually (23).

In Iran, 30-40 thousands disabled children are born every year (24) and excessive amounts of money are spent on the medical treatment and care of these children. Considering the suffering nature and psychological damages caused by such diseases, it is highly recommended to seek a logical solution to this problem. Genetic counseling can be very helpful in this matter. Counseling can be done either before or after genetic testing, the aim of which is to empower individuals to deal with different situations and help them to make appropriate decisions. Numerous ethical issues have been raised in the field of genetic counseling. In this regard, counselors should observe the general principles of medical ethics including respect for autonomy, beneficence, non-maleficence and justice. Considering the importance of ethics in genetic counseling, a survey of the ethical issues associated with clinical consultation in consanguineous marriage was a major part of this study. Obviously, genetic counseling without observance of ethical principles may be more harmful than beneficial. In the present study, ethical issues were investigated by presenting 3 clinical cases. In addition, to evaluate the clinical dimensions of the subject, the frequency of consanguineous marriages in clients of genetic counseling centers in Isfahan was studied.

1. Alnahaye, Ebn-e-Asir, vol. 3, p. 106; AlmojazatAlnabawi, Sharif Razi, p. 92.
2. Masalek Alafham, Shahid Thani, vol. 2, p. 38; Almhjh al-Bayda, Feiz Kashani, vol. 3, p. 94.

## Method

This study was conducted in two distinct parts including a cross-sectional descriptive design and a library research. Data collection tool was Form Number 1 prepared by the Deputy of Cultural Affairs and Prevention of the State Welfare Organization entitled “*The plan for the prevention of disabilities originating from genetic disorders*”. The questionnaire included demographic data, type of couples’ relationship, and history of family disorders in the family tree. The forms were completed by two genetic counselors (first and second authors of the present study) over the course of 5 years in the genetic centers of Isfahan, a large province in the center of Iran. The sample population consisted of all the couples who referred to genetic counseling centers in Isfahan during the years 1387 - 1392 (Persian solar year corresponding to 2008 - 2013 A.D.). The total sample size was 3,200 individuals. The data were analyzed by a statistician using the SPSS software version 16. The second part of the study was a library research using available references and books and examined the ethical challenges in the field of genetic counseling of consanguineous marriages. We searched the sources of Google as well as PubMed and Scientific Information Databases (SID) and Iran Medex using keywords such as “genetic counseling” and “consanguineous marriage” in combination with the word “ethic*” (in Farsi as well as English). Subsequently, related articles were selected on the basis of the abstract and sometimes the main text. The available books were also searched manually in the medical ethics department and library of the Endocrinology and Metabolism Research Institute of Tehran University of Medical Sciences. The overall content achieved by studying and analyzing these resources is presented in the results section.

***Ethical Considerations:***

The present study was approved by the Research Ethics Committee of the Endocrinology and Metabolism Research Center of Endocrinology and Metabolism Clinical Sciences Institute of Tehran University of Medical Sciences under the code EC-00327. The data resulting from this cross-sectional descriptive study were collected in genetic centers in Isfahan. Clients voluntarily referred to these centers for medical consultations about hereditary diseases. Their information was recorded in the questionnaire with their consent and used anonymously in the study. In the library research, we used the resources with honesty and integrity, and carefully observed the “National Ethical Guideline of Scholarly Publications in Medical Sciences”.

## Results

As mentioned above, this study consisted of two separate parts, and the results of each part will be presented below separately. 

*Part One: Determining the frequency of consanguineous marriages in the records of genetic counseling centers in Isfahan*

In this part, the medical documents of all couples who had referred to genetic counseling centers in Isfahan during the years 2008-2013 were reviewed. The total sample was 3,200 medical documents, of which approximately 130 samples were removed due to being incomplete. Therefore, the number of study samples reached 3070 people (1535 couples).

There were non-consanguineous marriages in only 25.7% of the cases. Among the consanguineous marriages, 62.3% of the cases pertained to third-degree relatives (first cousins)1, 9.1% to fourth-degree relatives (second cousins)2, and 1.9 % to fifth-degree relatives (third cousins)3. In addition, 1% of the participants were double cousins 4(Table 1). In total, 74.3% of the clients had consanguineous marriages, and a minimum of one hereditary disease was observed in the families of 75.3% of these individuals.

**Table 1:** The types of family relationships in the consanguineous marriages in this study

**Type of Family Relationship**		**No.**	**Percent (%)**
Non-Consanguineous Marriage		788	25.7
Consangu ineous Marriage	First cousins	1913	62.3
	Second cousins	279	7.8
	Third cousins	59	1.9
	Double cousins	31	1
	Total	2282	74.3
Total		3070	100

1. First cousins marriages: the marriage between the son and daughter of the maternal aunts, the son and daughter of parental uncles, maternal uncle's daughter and maternal aunt's son, parental aunt's daughter and maternal uncle's son.
2. Second cousins marriages: the marriage between uncle's grandchildren, maternal aunt and parental uncle's grandchildren, and grandchildren of maternal aunts.
3. Third cousins marriages: The marriage between great grandchildren as non-consanguineous marriage has low genetic risk; but when some genetic disorders exist in the family, this distant relationship may lead to diseases in children.
4. Double cousins marriages: The marriage in which there are two common ancestors, and the male and the female from both paternal and maternal families are close has the greatest risk.

**Diagram 1:**
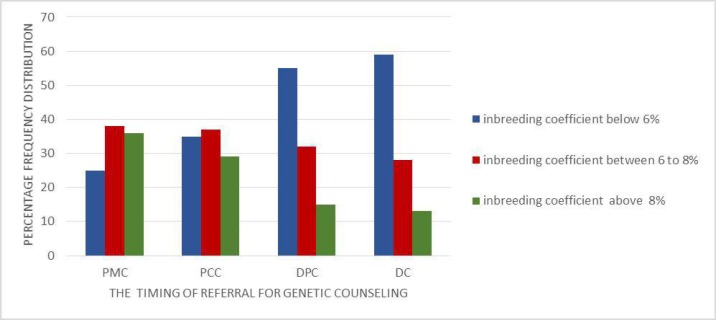
Clients’ inbreeding coefficient for the times of referral for counseling

As shown in Diagram 1, the inbreeding coefficient was various according to the time of referral for a broad range of counseling, including genetic counseling, pre-marriage counseling (PMC), preconception counseling (PCC), during pregnancy counseling (DPC) and diagnostic counseling (DC) of couples accompanied by their disabled children.

*Part Two: Library research on the ethical issues of clinical consultation in consanguineous marriages*

In recent years, pre-marriage counseling has been increasingly popular among couples who have records of certain genetic diseases in their families, or those who are planning consanguineous marriages. This is mainly due to the progress made in medical sciences and technologies on one hand, and the development of some methods of public education on the other. In fact, providing counseling services is mainly considered as a preventive approach to reduce defects and hereditary diseases. Pre-marriage genetic counseling is, however, complicated by numerous ethical issues originating from the strong emotions associated with marriage, and the critical decision-making related to spouse and fertility. The most important ethical challenges in this regard are mentioned below:

      *1. *    *Respect for autonomy and the couples’ informed decision making *

The importance of pre-marriage genetic counseling is undeniable, and if couples fail to have consultation, they may face adverse consequences that are sometimes costly. Nevertheless, according to the ethical principle of “autonomy”, any pressure by the government, community or physicians on couples and families to make them seek genetic counseling is impermissible (25). Autonomy is defined as respect for human beings’ inherent rights and their capacity for thinking, and the idea that they can make their own decisions and act on the basis of their values and beliefs (26). Therefore, it is recommended to encourage couples to attend consultations voluntarily through strategies such as public education, establishing conventional or distance workshops, and facilitating the process of counseling. During consultations, the necessity, goals, steps, benefits and even possible consequences of genetic counseling (such as overturning the decision to marry because of increased risk of developing genetic diseases in the offspring) should be clearly explained (27). Moreover, individual autonomy should be fully respected at all stages of consultation, including providing the information, screening, announcing the results, ensuring confidentiality, offering possible treatments, and the use of couples’ data in future studies. 

Respect for individuals’ autonomy means that they should receive the necessary information about the tools and available choices, and also be supported in making decisions based upon their personal authority while their questions and concerns are addressed (26). Sometimes the results of the tests and genetic screenings are inconclusive. In such cases, ethical appraisal should be carefully carried out in order to weigh correctly the couples’ autonomy in decision-making against the possible consequences (28). In other words, clinicians should not feel burdened with responsibility to force the couples to overturn their decision to be married. The issue of informed consent is critical in genetic counseling, as in many other fields of medical practice, and therefore it is necessary to consider the related principles and rules. Clients should also receive information about the possible benefits and harms of consanguineous marriage, which will enable them to make decisions voluntarily (26).

      *2. *    *Benefit and harm assessment of consanguineous marriage considering couples and the entire community*

Providing pre-marriage counseling has undeniable effects not only on the couples, but also on their families, the society, and even the future generations (29).

There are potential personal, social, professional and financial harms associated with genetic testing. Personal harms include anxiety, feelings of inefficiency and depression, changes in the goals and plans of life, and refusing treatment and medical care resulting from uncertainty in the genetic testing results (30). Some social harms consist of stigmatization, or manifestations of the privacy violation resulting from the propagation of the news of having a genetic disorder. While one example of professional harm is difficulty in employment (31), financial harms comprise high costs of genetic testing and sometimes long-term medical follow-ups (30). Considering all the issues mentioned above, effective measures should be taken to minimize the harms.

      *3. *    *Preserving confidentiality of families in pre-marriage counseling and its limits*

Some of the genetic testing results may impact the health of other family members, so confidentiality is rather important (32). It is necessary to provide couples with detailed information about the potential problems prior to performing diagnostic tests, and they should be well informed in case the identified genes may affect the health of other relatives. As ethical principles necessitate, the couple should be the first to learn about the results, and only with their consent may others be notified. In these cases, the couples’ confidentiality should be protected and only the most essential information may be disclosed (33).

Any attempt to inform other individuals who are at the risk of genetic harm against the couples’ wish is an ethical challenge that must be addressed prudently (34-36). In the meantime, willingness and autonomy of those individuals that are at high risk in case they do not receive genetic incompatibility information is an important factor that should be taken into account (37). 

      *4. *    *Professional ethics in the manner of disclosing information to couples and their families*

Professionals who conduct counseling sessions must consider several issues including (but not limited to) age and level of education, since these factors are crucial to determining the quantity and quality of information transfer (38). Culture and ethnicity also play an important role (39). The counselor should be well informed about the strategies, amount of information, and the audience to whom he/she wants to disclose genetic information (35). Other professional ethical principles to be observed by genetic counselors are truth telling and honesty, and avoiding paternalistic leadership. In fact, it is extremely important to provide information and counseling without any sort of judgment (28).

Specific skills are required in order to best manage clients’ psychological and emotional reactions (35, 40). Some ethical conflicts that consultants may face include: marriage counseling for only research and scientific purposes without any results for the clients, diagnostic failures and their consequences, conflicts and disagreements arising from value differences among consultants, clients, and their relatives (35), and also confronting clients under legal age (41). In each of these cases, the consultant has an ethical obligation in the manner of providing information to clients. Some genetic test results may necessitate the couples’ follow-up till they decide to have babies. In such cases, other ethical issues arise, for instance respect for confidentiality, relationship limits between consultants and clients, and conflicts of interest. Therefore, consultants should inform and assure couples that counseling facilities are always accessible for them and their families (42).

      *5. *    *Ethical principles regarding the use of clients’ data in research*

Due to the fast advances in genetic sciences and its effective role in many scientific fields such as medicine on one hand, and the costly nature of genetic testing on the other, data are utilized by researchers to ameliorate research projects. Thus, clients should be informed and receive a written informed consent. The fundamental point is that data owners should be well informed about the importance of genetic information. In addition, information preservation entails important considerations concerning the protection of confidentiality, for example why, when, how, by whom or in what electronic system the information will be stored (30), and who will have access to the data? On the same bases, biological and genetic databases generally include specific rules about issues such as identifiable or non-identifiable samples, long-term or short-term data storage, and performing numerous studies (43).

Another challenge arises in terms of using the data for future study purposes, bringing up questions such as in what conditions the owner of the samples should be informed about the new results of the studies (in order to gain profits and reject harms), and several other questions.

In this regard, announcements and ethical guidelines have also been developed at national and international levels. Following the approval of the* “Universal Declaration on the Human Genome and Human Rights*” (44) by UNESCO in 1997, the “*International Declaration on Human Genetic Data*” (45) was also approved in 2003. In Iran, the “*National Ethical Guidelines for Genetic Research*” was developed in 2004 along the same line (46). It is obvious that application of these guidelines will make a significant contribution to the ethical process of research in genetics.

      *6.*    * Justice in couples’ access to necessary counseling services*

In one sense, justice means that services need to be equally divided among individuals (30). As a matter of fact, in our country, all couples who want to have consanguineous marriage, or have a history of hereditary diseases in family, should have access to genetic counseling services in a fair and low-cost manner. Some developed countries provide free genetic counseling services and genetic testing, while in developing countries these services are expensive and are not widely available.

In this regard, the principle of equality and dignity of all human beings should be respected. Therefore, all people should have fair access to counseling services (47). In this vein, counseling services should be perfectly and fairly applied through creating suitable infrastructures and removing obstacles. The barriers to access genetic counseling services include: lack of adequate education and training of medical genetic professionals, lack of genetic counseling centers in the government sector, and lack of adequate insurance coverage (25).

There are important financial restrictions such as the costs imposed by diagnostic genetic laboratory tests, which can be a preventive factor in the couples’ decision to receive genetic counseling (48). In this regard, counselors should consider the necessity and priority of diagnostic tests, and should provide them with the best recommendations. Moreover, the issue of conflict of interests (i.e., directing individuals towards laboratories that bring financial profit for the consultants) is highly challenging and unethical at times (49).

      *7.*    * The intellectual property of innovative scientific results*

Sometimes investigating a rare disease leads to the discovery of a new gene or new mutation. The intellectual property of results and probably commercial profits arising from it may or may not belong to couples who have been tested. The issue should therefore be considered and determined before doing research, and the rights of each member of the research team must be respected by the principal investigator. 

***Clinical Cases***

Before proceeding with the discussion, we contemplate some clinical cases in order to pay more attention to the ethical challenges faced by practitioners in this field.

*The first case*: A 23-years-old woman and a 29-years-old man with the kinship of uncle’s grandson (fifth-degree) were advised to seek genetic counseling before marriage. Before the counseling session, the young man’s mother declared in a face to face and private contact that the family had not approved the marriage and failed to prevent him from it because of their son’s persistence. She then went on to insist that the practitioner prohibit the marriage regardless of the results of the genetic counseling and tests.

*The second case* **: **A young couple with distant kinship or relationship (above fifth-degree) referred to a genetic clinic before marriage. There were no signs of hereditary diseases in their family tree. After a few months of marriage, the woman’s mother visited the counselor alone and stated that three uncles of the bride were paralyzed! She requested from the genetic counselor a letter stating that the condition could be transferred to the couple’s future children and that they would definitely be affected.

*The third case* **: **A 25-years-old woman was planning a consanguineous marriage with her 28-year-old cousin (her uncle’s son). There were no records of genetic diseases in the family tree, but a genetic counselor had requested genetic tests in the previous session. The couple had not done the tests yet, and since they had been referred to a private center (where the previous counselor happened to be a stakeholder), they would have to bear enormous costs!

## Discussion

Consanguineous marriage is a complex and multi-faceted issue, and no major, successful action has been taken to control it so far, mainly due to a lack of consideration for cultural, social and geographical variables. A review of the texts reveal that in societies where consanguineous marriage is common and fostered by the existing customs and traditions, people’s awareness of the harms is very low. Couples, especially those who are married at a younger age, do not have the necessary knowledge in this field, and mass communication has seemingly failed to educate the public on the issue. Therefore, encouraging couples to perform genetic counseling before marriage, raising their level of knowledge during the sessions, and answering their questions can be helpful in carrying out a scientific and rational decision.

In the present study, which was performed on couples who had referred to genetic counseling centers in Isfahan, about 75% of the marriages were consanguineous, and more than 62% of the clients were close relatives. It should be mentioned that the study was conducted on clients of counseling centers, not all the couples who had been married in that period of time. The current study results cannot be generalized to the entire community, but can indicate the importance of the issue in the country. In a similar study conducted on 2686 couples who had referred to genetic counseling centers in Shiraz during a four-year period, 85% had consanguineous marriages, 23% of whom were first cousins (50). Furthermore, in a study that was conducted in Tabriz over a period of 4 years, 20.12% of the marriages were consanguineous, more than 50% of which were between first cousins (51). In another study on the various ethnic groups from different regions of Iran, 38% had consanguineous marriages, 70% of whom were first cousins (52).

One limitation of the study was that we could not provide exact statistics on the prevalence of consanguineous marriage, because the clients referring to genetic counseling centers in Isfahan are not representative of all the couples in the community.

While considering the issues mentioned in the results section facilitates the ethical process of genetic counseling, it seems that studying the psycho-social consequences of consultations is also necessary. Such investigations will help to determine the method of counseling by clarifying the social and psychological effects and can reduce the undesirable consequences. In addition, genetic counseling training programs can make genetic specialists aware of how effective the ethical principles will be. Conscience may also serve as a moral gauge for decision-making in difficult situations. Medical geneticists’ collaboration with medical ethics experts and referring specific and controversial cases to them may also play an effective role in resolving challenges.

During genetic counseling, the autonomy of families should be respected, and it must be noted that the unconscious choice will lead to regret. Genetic counseling before marriage should be done in the presence of couples’ parents, because consanguineous marriage is usually proposed by one of the parents, and if the genetic counselor can convince the elders, couples will be able to make a better decision in a more relaxed atmosphere far from family coercion. Respect for elders and their beliefs and customs and providing them with related scientific research results and past experiences can always be helpful.

In pre-marriage counseling sessions, couples state their problems privately and genetic counselors should ensure confidentiality. Preserving the ethical commitment towards clients can reduce family conflicts. In some cases, there are dangerous inherited diseases among the family members of a client that he/she may not wish to reveal in the genetic counseling session. In such cases, ethical responsibility forces the counselor to persuade the client to provide the necessary and true information to his/her future spouse and then consult the genetic counselor about strategies to prevent those diseases from being transferred to the offspring.

***Discussion of clinical cases***

*The first case (a real case):* Considering the principle of confidentiality and the potential profits and harms, the genetic counselor drew the family tree in full honesty and explained the inbreeding coefficient to the couple. They had a fifth-degree consanguineous marriage and an inbreeding coefficient of 4% was determined as the incidence risk of disease in their children. The counselor explained that even if the couple’s kinship is distant, the incidence risk of diseases in children will still be higher in consanguineous marriages than non-consanguineous ones. The counselor also emphasized that no genetic testing and paraclinical measures could give full confidence to the couple that their child would be born healthy. Finally, according to the principle of respect for individual autonomy, decision-making was left to the couple and their families. 

*The second case (a real case):* In pre-marriage genetic counseling, families are provided with comprehensive information, and therefore in the case of consanguineous marriages, a full explanation is provided based on the risk of certain diseases in children. The situation is different in genetic counseling after marriage, since the family cannot be disintegrated on the pretext of disease risk in children. In this case, the couple had a distant relationship and the disease was recognized only after the marriage. Therefore, the genetic counselor advised members of the family who suffered from the disease to have genetic testing so that in the case of a definite diagnosis of gene mutation, carrier testing could be done for the couple. In the case of carrier couples, fetal sampling during pregnancy and prenatal diagnosis should be provided, and in the case of diseases that lead to severe disabilities in children, after obtaining the family’s consent and the approval of forensic medicine authorities, an abortion permit can be issued. Thus, being honest with couples and their families is beneficial in such cases. 

In diagnostic genetic counseling, it should be notified that sometimes a disease is transferred by one of the parents to the child. The facts cannot be stated unless the couple requests investigation of the cause of the disease, and any suspicion and non-scientific speculation in this regard should be avoided. Only after genetic testing and establishment of a definitive reason can the issue be shared with the couple. In these cases, even informing their families of the existence of the disease should be done only by the couple or with their consent. 

*The third case (a real case):* Financial problems can greatly impact the relationship between the counselor and clients. Considering the patients’ benefits and harms and avoiding the imposition of unnecessary costs are moral imperatives. To avoid conflict of interests, counselors must not refer their clients to facilities that they own, since that would be an unethical practice, and in some countries could even result in heavy punishment.

There is a misconception that by having genetic testing a couple can be assured of their child’s health despite consanguineous marriage. There are ethical concerns in this regard that are considerably important. It is unethical to encourage families to do unnecessary tests and then assure them of the test results. The genetic counselor should clearly notify couples about the high incidence of consanguineous diseases and the limited predictive value of the tests. Sometimes families pay enormous sums on genetic testing and think that favorable results mean that the risks associated with consanguineous marriage have been removed completely! Unfortunately, such an assurance is given to couples in some counseling centers for reasons such as financial gain and commercial arrangements. Professionalism in the field of genetic counseling necessitates that testing be done only in helpful situations. 

## Conclusion

Genetic counseling is known as one important way to prevent congenital diseases. Providing genetic counseling plays a special role in decision making capacity building for important issues such as choosing a spouse and fertility. Genetic counseling also presents many ethical challenges for professionals. Therefore, it is necessary for experts in this field to be familiar with the related ethical issues and the principles of ethical decision-making.

One basic principle in the health system is the priority of prevention to treatment, and the authorities need to provide practical and effective ways to reduce costs and provide equitable access to genetic counseling facilities. Indeed, the ethical process of these consultations will have a significant impact on couples’ decision-making. Considering the statistics obtained in this study and the important ethical issues discussed throughout the paper, the following key points and recommendations can be offered:

- Applying comprehensive programs to prevent the birth of children with disabilities is a professional commitment in line with the principle of justice in distribution of health resources.
- Consanguineous marriage is deeply rooted in the culture of some ethnic and social groups. As an initial step, the society must be perfectly informed of its disadvantages and consequences in order to be able to act consciously in this regard.
- As a religion that pays attention to the worldly and spiritual matters of its followers, Islam has never encouraged consanguineous marriage, and has even prohibited it in some cases. This point can be used and emphasized in counseling.

Finally, providing specific courses on ethical issues related to genetic counseling is a necessity in Iran. Two main goals of such a course should include enhancement of ethical sensitivity, and empowerment of counselors to help them act according to ethical codes and make appropriate decisions when encountering ethical conflicts.
